# Metformin and Fibrosis: A Review of Existing Evidence and Mechanisms

**DOI:** 10.1155/2021/6673525

**Published:** 2021-04-29

**Authors:** Maoyan Wu, Huiwen Xu, Jingyu Liu, Xiaozhen Tan, Shengrong Wan, Man Guo, Yang Long, Yong Xu

**Affiliations:** ^1^Department of Endocrinology and Metabolism, The Affiliated Hospital of Southwest Medical University, Luzhou, Sichuan, China 646000; ^2^Cardiovascular and Metabolic Diseases Key Laboratory of Luzhou, Luzhou, Sichuan, China 646000; ^3^Sichuan Clinical Research Center for Nephropathy, Luzhou, Sichuan, China 646000; ^4^Southwest Medical University, Luzhou, Sichuan, China 646000

## Abstract

Fibrosis is a physiological response to organ injury and is characterized by the excessive deposition of connective tissue components in an organ, which results in the disruption of physiological architecture and organ remodeling, ultimately leading to organ failure and death. Fibrosis in the lung, kidney, and liver accounts for a substantial proportion of the global burden of disability and mortality. To date, there are no effective therapeutic strategies for controlling fibrosis. A class of metabolically targeted chemicals, such as adenosine monophosphate-activated protein kinase (AMPK) activators and peroxisome proliferator-activated receptor (PPAR) agonists, shows strong potential in fighting fibrosis. Metformin, which is a potent AMPK activator and is the only recommended first-line drug for the treatment of type 2 diabetes, has emerged as a promising method of fibrosis reduction or reversion. In this review, we first summarize the key experimental and clinical studies that have specifically investigated the effects of metformin on organ fibrosis. Then, we discuss the mechanisms involved in mediating the antifibrotic effects of metformin in depth.

## 1. Introduction

Fibrosis is a response in organs to a trigger or injury and is described as a condition with excessive deposition of connective tissue components in an organ. The deposition of extracellular matrix (ECM) proteins leads to the disruption of physiological architecture, organ remodeling, and ultimately organ malfunction. Diseases including nonalcoholic steatohepatitis (NASH), nonalcoholic fatty liver disease (NAFLD), cirrhosis, chronic kidney disease, heart failure, myocardial infarction (MI), diabetes, idiopathic pulmonary fibrosis (IPF), and scleroderma are strongly associated with fibrosis. In total, the annualized incidence of major fibrosis-related conditions is approximately 4968 per 100,000 person-years. Furthermore, the global burden of liver fibrosis is significant, affecting 1 in 4 people [1, 2]. The prevalence of fibrosis is 1 in 6 persons for the kidneys, 1 in 60 for the heart, 1 in 1500 for the lungs, and 1 in 5400 for the skin [3–8].

**Figure 1:**
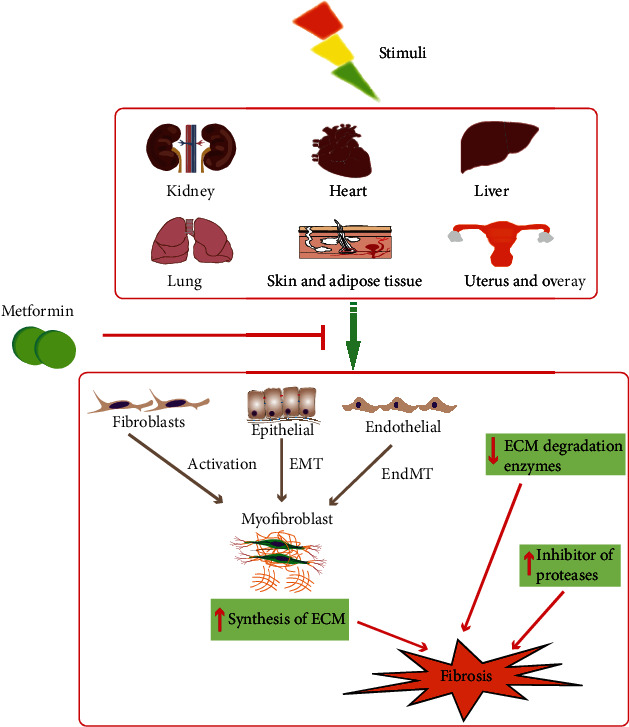
Metformin and organ fibrosis.

**Figure 2:**
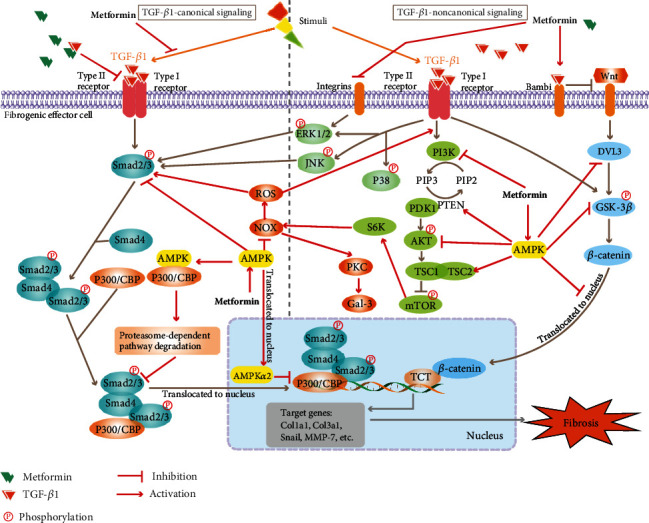
The canonical and noncanonical TGF-*β*1 signaling pathways in metformin-mediated antifibrotic effect.

**Figure 3:**
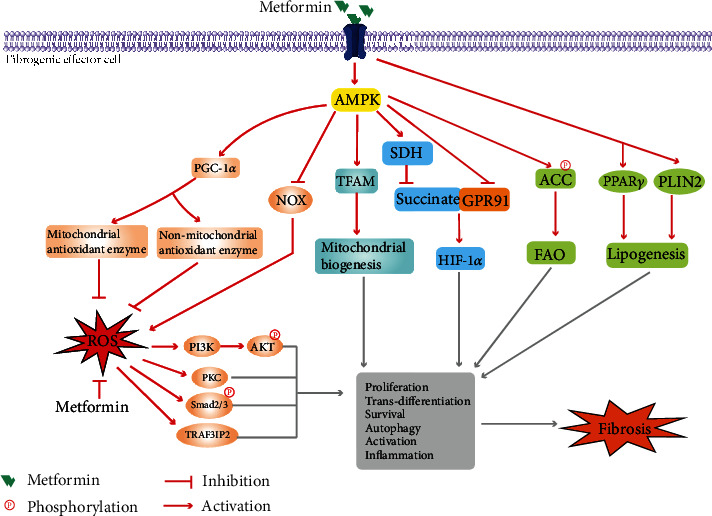
The other potential mechanisms of metformin in antifibrosis.

The outcomes of fibrosis depend on the affected organ. Fibrosis of internal organs, such as the liver, kidney, heart, or lung, leads to organ dysfunction and failure and finally to death. However, there is currently no effective cure for fibrosis. It is meaningful to identify means of ameliorating the progression of tissue fibrogenesis. Metformin was initially developed as an antidiabetic drug in the 1950s and has gained further attention due to its many potential therapeutic benefits, excellent safety profiles, and relatively low risk of side effects [9]. Owing to its excellent performance in controlling glycemia, as well as its cost-effectiveness and safety, it is recommended as the only first-line drug for the treatment of type 2 diabetes (T2DM). Additionally, metformin also exerts weight loss [10], antioncogenic [11], anti-inflammatory [12], and antiaging [13] effects and influences the gut microbiota and immune system [14]. Interestingly, emerging experimental data from *in vivo* and *in vitro* studies indicate that metformin performs excellently in fighting fibrosis. It is suggested that metformin may have potential in clinical therapy, and some clinical trials have evaluated its potential in treating fibrosis in patients with liver, lung, and ovarian fibrosis. As mentioned above, metformin is a versatile drug that can act on multiple organs and tissues through a variety of potential mechanisms [15]. Most importantly, the liver and muscles are the main targets of metformin, which effectively lowers glucose by increasing glucose uptake in the liver and muscles and reducing gluconeogenesis. Metformin has been reported to inhibit intestinal glucose absorption, enhance intestinal glucose utilization, improve glucagon-like peptide-1 (GLP-1) secretion from intestinal enteroendocrine L cells, and modulate gut microbiota [16, 17]. Mitochondrial respiratory chain and adenosine monophosphate-activated protein kinase (AMPK) activation may play a central role in metformin-mediated regulation of energy metabolism and the redox state. Anti-inflammatory effects and modulation of immune cell function have also been reported. By searching the literature, we found that there are many studies on the mechanisms of metformin in fibrosis, which indicate that metformin mainly exerts antifibrosis effects by affecting the Transforming Growth Factor Beta (TGF-*β*) signaling pathway, cell metabolism, and oxidative stress. In this review, we first summarize the key experimental and clinical studies that have specifically investigated the effects of metformin on organ fibrosis (shown in Figure 1). Then, we discuss the mechanisms involved in mediating the antifibrotic effects of metformin in depth (shown in Figures 2 and 3).

## 2. Experimental and Clinical Evidence of the Antifibrotic Effects of Metformin

Metabolic alterations are increasingly recognized as important in the pathogenesis of fibrosis in many organs. Metformin, which is a potent AMPK activator and is effective in ameliorating insulin resistance, has emerged as a promising method to fight against fibrosis.

Recently, a study published in Nature Medicine aroused wide interest. In this study, researchers found that metformin treatment effectively prevented and slowed the progression of fibrosis, and impressively, it was potent in promoting fibrosis resolution and reversing established fibrosis in a bleomycin-induced murine lung fibrosis model [18]. Consistently, positive outcomes were also observed in bronchopulmonary dysplasia induced by hyperoxia [19], PM2.5-induced lung injury [20], cultured precision-cut lung slices derived from human IPF patients [21], and radiation-induced pulmonary fibrosis [22]. Some studies also found that metformin administration improved left ventricular function in a permanent left coronary artery occlusion mouse model [23] and in dogs with pacing-induced heart failure [24]. Metformin-mediated cardioprotective effects may be attributed to significantly reduced cardiac fibrosis induced by rapid right ventricular pacing [24], pressure overload [25], Ang II [26, 27], *δ*-sarcoglycan deficiency [28], MI [29], and PM2.5 [20]. Additionally, metformin treatment has been demonstrated to slow cystogenesis in a murine model of autosomal-dominant polycystic kidney disease [30]. It has also been reported to reduce renal fibrosis in high-fat diet-fed rats [31], unilateral ureteral obstruction (UUO) mice [32, 33], folic acid-induced renal fibrosis [32, 34], adenine-induced chronic kidney disease rats [35], and cyclosporine A-induced renal fibrosis rats [36]. Moreover, accumulating evidence has shown that metformin has great potential in alleviating the progression of fibrosis in various organs. Metformin produced antifibrotic effects and protected against aberrant ECM remodeling in visceral adipose tissue of ob/ob mice [37] and subcutaneous adipose tissue of doxorubicin-treated mice [38]. Metformin-mediated reduced fibrosis was also observed in ovarian and uterine tissues from a dehydroepiandrosterone-induced polycystic ovarian syndrome model [39], skin of bleomycin-induced scleroderma [40], peritoneal tissue of a peritoneal dialysis animal model [41], and peritendinous tissue of injury-induced peritendinous adhesion rats [42].

Clinically, it has been proven that metformin therapy results in isotropic collagen organization and reduced fibrosis in postmenopausal ovaries of patients taking metformin for T2DM treatment at the time of oophorectomy [43]. Given the ovarian cancer risk reduction observed with metformin use in T2DM women [44] and the role of metformin in suppressing age-associated fibrosis in ovaries, metformin is suggested to have potential for ovarian cancer prophylaxis. Consistently, an open-labeled, randomized trial in which 55 nondiabetic NAFLD patients were treated with metformin at a daily maximum dose of 2000 mg/day for 12 months showed that metformin treatment produced more than decreased fat percentage and necroinflammation, as it was surprising that a remarkable decrease in fibrosis score was observed in the posttreatment biopsy of 17 metformin-treated NAFLD patients [45]. It was disappointing that some clinical trials unexpectedly showed that metformin treatment was less effective in ameliorating liver fibrosis in NAFLD patients [46–51]. Despite the lesser effects of metformin on liver fibrosis, it was shown that metformin effectively restricted damage to hepatocytes with decreased levels of alanine aminotransferase (ALT) and aspartate aminotransferase (AST) and resulted in improved necroinflammation and ballooning degeneration scores [47, 48, 51].

As mentioned above, *in vitro* and preclinical experiments showed how strong and potent metformin is in fighting fibrosis. However, evidence from clinical studies on the antifibrosis effect of metformin is insufficient. Next, we will discuss the mechanisms involved in mediating the antifibrotic effects of metformin in depth based on existing research, summarize the targets of metformin's antifibrosis effects, and try to provide directions for future clinical trials of metformin's antifibrosis effects.

## 3. The Underlying Mechanisms of Metformin in Antifibrosis

### 3.1. Metformin and TGF-*β*1 Signaling Pathways

#### 3.1.1. Canonical TGF-*β*1 Signaling Pathways

Activation of the TGF-*β*1-Smad2/3 cascade plays an essential role in gene expression, including encoding ECM proteins. Activated TGF-*β*1 binds to its type II receptor, which recruits its type I receptor and then phosphorylates the COOH-terminal domains of Smad2 and Smad3. Phosphorylated Smad2 and Smad3 form a complex with Smad4 to translocate to the nucleus and transcriptionally activate fibrogenic target genes such as collagen 1*α*1 (col1a1) and collagen 3*α*1 (col3a1) [42, 52]. Most studies have shown that metformin directly exerts antifibrotic effects by inhibiting TGF-*β*1 production and subsequently decreasing the phosphorylation and nuclear translocation of Smad2/3 [25, 34, 53–55]. Additionally, some studies have found that metformin exerts antifibrotic effects by blocking the phosphorylation of Smad2/3 [22, 42, 54, 56]. Furthermore, metformin treatment and subsequent AMPK activation inhibited the phosphorylation and nuclear translocation of Smad3 [37]. Decreased reactive oxygen species (ROS) generation induced by metformin treatment modulates TGF-*β*1-induced Smad2/3 phosphorylation and myofibroblast differentiation [41, 57]. ROS-sensitive regulation of tyrosine kinases and protein tyrosine phosphatases accounts for a decrease in Smad2/3 phosphorylation [58]. Moreover, metformin has also been shown to directly interact with TGF-*β*1 at its receptor-binding domain, thus suppressing the binding of TGF-*β*1 to its receptor and resulting in decreased activity of downstream signaling [59]. However, some studies reported that metformin did not reduce TGF-*β*1-stimulated Smad3 phosphorylation but inhibited TGF-*β*1-stimulated gene transcription driven by Smad3 [60–62]. P300/CREB-binding protein (CBP), which is a coactivator with intrinsic acetyltransferase activity, often cooperates with Smads to regulate the transcription of target genes and plays a pivotal role in the fibrotic responses of various cell types. Upon TGF-*β*1 stimulation, the interaction of Smad2/3 with P300/CBP induces acetylation in the N-terminal region of Smad2/3, which results in Smad-dependent gene transcription [63–65]. Activated AMPK induced by AICAR or metformin competes with Smad3 for interaction with P300 and targets it for degradation through a proteasome-dependent pathway [61]. This AMPK-dependent degradation of P300 and decreased interaction between P300 and Smad3 accounts for the decreased acetylation and transcriptional activity of Smad3, which inhibits the TGF-*β*1-induced fibrogenic property of hepatic stellate cells (HSCs). Additionally, active AMPK blocks Smad3-mediated transcription by promoting the translocation of AMPK*α*2 to the nucleus without inhibiting Smad3 phosphorylation or nuclear translocation [62].

#### 3.1.2. Noncanonical TGF-*β*1 Signaling Pathways

*(1) MAPK Signaling Pathways*. In addition to canonical Smad-mediated transcription, TGF-*β*1 also activates other noncanonical signaling cascades, including mitogen-activated protein kinase (MAPK) pathways, which mediate a variety of cellular responses, including cell cycle control, apoptosis, and differentiation [66]. MAPKs are a family of serine-threonine protein kinases, the most extensively studied of which are extracellular signal-regulated kinase 1 and 2 (ERK1/2), c-Jun N-terminal kinase (JNK), and p38 kinases [66]. MAPK activation results in serine/threonine phosphorylation in the linker region or MH1 domain in R-Smads, leading to transcriptional activation of the collagen promoter and enhancement of collagen synthesis [52, 67, 68]. Some studies reported that metformin treatment resulted in inhibition of the phosphorylation and activity of ERK1/2, which is an important mechanism in preventing fibrotic progression [33, 34, 41, 42, 54, 55, 69–71]. AMPK-mediated inhibition of ERK1/2 phosphorylation blocked cell cycle progression and decreased the number of S- and G2/M-phase fibroblasts, leading to inhibition of fibroblast proliferation, migration, and differentiation [42, 72]. Integrin is an important cell receptor that mediates ECM remodeling and plays a vital role in regulating the ERK signaling pathway. Metformin treatment can modify integrin expression, decrease ERK1/2 phosphorylation, and ameliorate the expression of ECM components [73]. Furthermore, metformin administration significantly inhibits TGF-*β*1-induced monocyte chemotactic protein-1 expression through BMP and activin membrane-bound inhibitor- (Bambi-) mediated suppression of MEK/ERK1/2 signaling in cultured rat renal tubular epithelial cells [74]. Moreover, activation of p38 MAPK and JNK is involved in the fibrosis process [66]. Metformin treatment could also decrease the activities of p38 MAPK and JNK, contributing to fibroblast resistance to apoptosis and aggravation of fibrosis [55, 75]. However, inconsistent with these studies, metformin administration did not change the activity of AMPK and p38 MAPK or the collagen levels in TGF-*β*1- or high glucose-treated cardiac fibroblasts [76].

*(2) Wnt Signaling Pathways*. Wnt signaling pathways, which were initially discovered as major regulators of carcinogenesis and embryonic development, have been reported to play critical roles in regulating fibrotic progression. Activation of canonical Wnt/*β*-catenin signaling phosphorylates and inactivates GSK-3*β* and results in mobilization of *β*-catenin into the nucleus, where it promotes the transcription of various fibrogenic genes, including fibronectin, Snail, and matrix metalloproteinase-7 (MMP-7) [77–79]. Metformin blocked the effect of TGF-*β*1 on the phosphorylation of GSK-3*β* and nuclear translocation of *β*-catenin and subsequently suppressed the transcription of Snail and MMP-7 [41]. This metformin-mediated inhibition of the Wnt signaling pathway contributes to restoring the expression of E-cadherin and reversing the epithelial-mesenchymal transition (EMT) induced by TGF-*β*1 in human peritoneal mesothelial cells. Furthermore, metformin impedes the canonical Wnt axis and deregulates Wnt-regulated gene expression by directly inhibiting the expression of DVL-3 (a Wnt mediator), leading to delayed onset of EMT and impaired neural crest development [80]. It has also been reported that metformin inactivated GSK-3*β* by enhancing the activity of protein kinase B (PKB, also known as AKT) and then alleviated cardiac fibrosis in STZ-induced diabetic rats [75]. In addition, metformin has been observed to upregulate TGF-*β* pseudoreceptor Bambi expression and to promote the survival of quiescent HSCs by activating the Wnt signaling pathway [81].

*(3) PI3K/AKT Signaling Pathways*. Phosphatidylinositol 3′-kinase (PI3K)/AKT is a critical signaling pathway in regulating glucose metabolism, proliferation, apoptosis, and differentiation. It is involved in regulating the TGF-*β*1 pathway and plays an important role in regulating fibrosis by promoting the activation and proliferation of fibroblasts and ECM synthesis. Metformin blocked the fibrogenic response of HSCs induced by platelet-derived growth factor (PDGF) by inhibiting the activities of AKT and its downstream target mammalian target of rapamycin (mTOR) [69]. Metformin treatment may attenuate the hyperglycemia-induced inhibition of the cardiac liver kinase B1/AMPK/AKT pathway, activate GSK3-*β*, and prevent diabetes-induced cardiomyopathy [75]. Decreased expression of phosphatase and tensin homolog deleted on chromosome ten (PTEN) and activation of the PI3K/AKT signaling pathway in the kidneys of diabetic nephropathy mice were reversed by metformin treatment, which may lead to decreased expression of miR192 [82]. Additionally, metformin-induced inactivation of the PI3K/AKT signaling pathway has also been reported as a consequence of AMPK activation and subsequent decreased NADPH oxidase- (NOX-) dependent ROS production [83]. Metformin administration upregulated the expression of the regulatory subunits of PI3K PIK3r1 and p53, which in turn suppressed forkhead box-O3 expression and ameliorated radiation-induced skin fibrosis in mice [84]. However, metformin administration decreased the expression of PI3K in the liver but not in the lungs of common bile duct ligation rats, and this effect of metformin on PI3K expression was independent of AMPK activation [85]. Furthermore, inconsistent with other studies, decreased activity of the PI3K/AKT signaling pathway and enhanced endoplasmic reticulum stress-induced apoptosis were observed in an intrauterine adhesion model, and metformin treatment reversed these changes and protected the endometrium from fibrosis after mechanical injury [70]. Restoration of the PTEN/PI3K/AKT signaling pathway and protective effects against myocardial fibrosis were also reported in metformin-treated T2DM mice [86].

*(4) mTOR Signaling Pathways*. mTOR is a highly conserved serine/threonine protein kinase in the PI3K-related kinase family and is well known to adjust fibrogenetic processes by regulating autophagy, inflammation, and fibroblast proliferation and differentiation [87]. mTOR activity has been reported to be reduced by metformin in an AMPK-dependent manner and to mediate antifibrotic effects [18, 29, 69, 88]. Upon AMPK-mediated mTOR inactivation, metformin treatment significantly enhanced mitochondrial biogenesis and reversed reprogrammed aerobic glycolysis, which in turn enhanced collagen turnover via autophagy, restored the cell sensitivity to intrinsic apoptosis, and finally accelerated the resolution of bleomycin-induced lung fibrosis [18]. Cardiomyocyte-secreted galectin 3 (Gal-3), which is a member of the *β*-galactoside-binding protein family, has been reported to mediate cardiac fibroblast activation and promote myocardial fibrosis [89]. Reduced expression of Gal-3 has been observed in cardiomyocytes in metformin-treated MI mice and led to ameliorated cardiac fibrosis after MI [29]. Decreased NOX4 activity and mitochondrial oxidative stress and inhibited protein kinase C *α* (PKC*α*) activity, which accounted for the effect of metformin on Gal-3 expression, may be a consequence of increased levels of TSC2 phosphorylation and subsequent inactivation of the mTOR-S6K signaling pathway [29]. However, metformin treatment did not show any effects on methionine-choline-deficient- (MCD-) induced increases in mTOR activity or the NASH phenotype, such as inflammation and collagen deposition, in NASH mice [90].

### 3.2. Metformin and ROS

ROS play an important role in regulating multiple cellular processes, including proliferation, differentiation, migration, and apoptosis. Oxidative stress, which is characterized by excessive ROS generation, is known to be a central mechanism involved in promoting fibrotic progression. AMPK is a suppressor of oxidative stress and is critical in regulating ROS production. As expected, metformin, as a defined AMPK activator, has also been observed to reduce ROS production and to suppress oxidative stress [29, 41, 55, 57, 83, 91–94]. Decreased expression and activity of NOX, especially NOX4, accounts for decreased ROS production induced by metformin [29, 41, 57, 83, 92, 94]. Metformin was also reported to decrease mitochondrial ROS production by protecting mitochondria from TGF-*β*1-induced damage and upregulating the mitochondrial antioxidant system [41]. Furthermore, metformin decreased ROS generation with enhanced antioxidant activity by reducing malondialdehyde (MDA) expression and the glutathione (GSH/GSSG) ratio and upregulating the expression of antioxidant enzymes, such as superoxide dismutase 2 (SOD2), heme oxygenase-1, and thioredoxin [41, 55, 83, 93, 94]. Peroxisome proliferator-activated receptor gamma coactivator-1*α* (PGC-1*α*), which is a versatile transcriptional coactivator, has been reported to be upregulated by metformin in an AMPK-dependent manner and to be involved in regulating the expression of the mitochondrial antioxidant enzyme SOD2 and the nonmitochondrial antioxidant enzyme catalase [83]. Reduced ROS production may interfere with the TGF-*β*1 signaling pathway by directly regulating Smad2/3 activity [57]. Metformin has also been reported to attenuate PDGF-induced intracellular ROS production and the subsequent PI3K/AKT signaling pathway. Additionally, reduced ROS-mediated PKC inactivation is involved in the effect of metformin on suppressing the activation and proliferation of myofibroblasts and reducing ECM production [29, 91]. Two isoforms of PKC, PKC*α* and PKC*ε*, have been reported as targets of metformin in its antifibrotic effect. Metformin treatment also suppressed oxidative stress-associated inflammatory factor release by downregulating the expression of the oxidative stress-response cytoplasmic adaptor molecule TRAF3 interacting protein 2 (TRAF3IP2), consequently reversing aldosterone+salt-induced cardiac fibrosis [92].

### 3.3. Metformin and Metabolism

Metabolic alterations, such as enhanced glycolysis and arginine metabolism, dysregulated fatty acid metabolism, and suppressed sphingolipid metabolism, have been observed during the pathogenesis of fibrosis and play key fundamental roles in regulating ECM homeostasis and fibrosis [95]. Reversing these metabolic alterations has emerged as a promising strategy to fight fibrosis.

Acetyl-CoA carboxylase (ACC), as a target for energy-sensing AMPK, is the major controller of the rate of intracellular fatty acid metabolism [96]. It has been observed that phosphorylation of ACC1 at Ser79 and ACC2 at Ser212, both of which respond to activation of AMPK and are associated with decreased catalytic activity, was associated with higher de novo lipogenesis and lower fatty acid oxidation (FAO), as well as increased levels of fibrosis in the liver [96]. Metformin therapy, which led to AMPK activation, increased phosphorylation of ACC1 at Ser79 and reduced lipid accumulation and fibrosis in the kidneys from a UUO model [32]. This AMPK/ACC-dependent antifibrotic effect of metformin was also confirmed in a carbon tetrachloride-induced liver fibrosis model and abrogated the development of hepatocellular carcinoma [97]. However, inconsistent consequences were observed in patients with IPF [21]. Similarly, metformin treatment accelerated fibrosis resolution in cultured lung tissue from patients with IPF and in a bleomycin-induced lung fibrosis mouse model. However, unlike the antilipogenic effect mentioned above, metformin triggered the transdifferentiation of myofibroblasts into lipofibroblasts by enhancing the activity and expression of lipogenic markers such as peroxisome proliferator-activated receptor gamma (PPAR*γ*) and PLIN2 and induced lipid droplet accumulation in human IPF fibroblasts. Interestingly, metformin-induced AMPK activation accounted for the suppression of myofibroblast marker COL1A1 expression but not the expression of lipogenic markers. These antifibrotic effects of metformin responded to upregulated BMP2 and PPAR*γ* phosphorylation.

Succinate, which is an intermediate metabolite of the Krebs cycle, is converted to fumarate by succinate dehydrogenase (SDH) and is recognized as an important signaling molecule in regulating ROS production, inflammation, fibrosis, etc. [98, 99]. Succinate accumulation has been reported in palmitate-stimulated HSCs and NAFLD mice induced by an MCD diet or a high-fat diet and is associated with HSC activation and NAFLD-associated liver fibrosis [100, 101]. Metformin therapy has been observed to reduce succinate accumulation by inhibiting the expression of SDH and to downregulate hypoxia-inducible transcription factor-1*α* (HIF-1*α*) expression and ameliorate liver fibrosis [100]. Along with decreasing succinate accumulation, metformin inhibited the expression of GPR91, which is the specific receptor of succinate [102], and subsequently ameliorated HSC activation and hepatic fibrosis [101].

### 3.4. Metformin and Mitochondrial Function

Many progresses of fibrosis, such as fibroblast activation, ECM synthesis, secretion, and degradation, require significant energy input, which is provided by the ATP generated from upregulated glycolysis and (FAO) [95]. It has been reported that metformin ameliorates high-fat diet-, palmitate-, and hypoxia-induced endoplasmic reticulum stress and HIF-1*α* expression and simultaneously inhibits adipose fibrosis. This may be attributed to decreased ATP production, limited oxygen consumption, and low oxygen tension and effectively prevented hypoxia in adipocytes [103]. A recent study reported that deficiency in AMPK activity, which was observed in fibroblasts from IPF, diminished basal oxygen consumption, the ATP-linked O2 consumption rate (OCR), maximal respiration, and the mitochondrial reserve capacity. Metformin treatment triggers mitochondrial biogenesis by upregulating mitochondrial transcription factor (TFAM) and induces the expression of the major components of mitochondrial ETC complexes, such as NDUFB8, 30 kDa FeS, core protein 2, C-IV subunit I, and the *α* subunit, in human lung fibroblasts [18].

## 4. Conclusion

Metformin has been an excellent first-line oral hypoglycemic agent for T2DM for many years. Its efficacy, safety, and cost benefits have been widely recognized. In addition to hypoglycemic effects, other potential indications for metformin have emerged. As mentioned above, metformin exerts potentially favorable effects in relation to the prevention and treatment of organ fibrosis. These properties have attracted an enormous amount of attention from researchers. Unfortunately, most of the above studies are preclinical, and to date, effective evidence of metformin as an antifibrotic agent in the clinic is insufficient. There may be several explanations for such a discrepancy. First, variations among different species may partly account for these inconsistent results. Different Smad proteins are involved in TGF-*β*1 signal transduction. Furthermore, a single mouse model cannot fully recapitulate the human disease state. Additionally, one main reason is that AMPK activation is only relevant to certain fibrosis phenotypes. Another reason for such a discrepancy in results may be due to the difference between doses used in experimental studies and those achieved in clinical trials. Such inconsistent data call for more in-depth, well-organized, randomized, and controlled clinical trials to investigate its efficacy in this indication.

In this review, we also discuss the mechanisms involved in the antifibrotic effect of metformin. As mentioned above, the canonical TGF-*β*1 signaling pathway is the main target pathway, and noncanonical TGF-*β*1 signaling pathways, such as the MAPK, Wnt, mTOR, and PI3K/AKT signaling pathways, are also involved. It has also been reported that metformin can ameliorate fibrosis by regulating mitochondrial function and improving oxidative stress. In these proposed mechanisms, AMPK activation plays an important role but is not dispensable in the antifibrotic effect of metformin (as shown in Table 1).

## Figures and Tables

**Table 1:** We use different symbols to indicate the different role of AMPK in these studies. AMPK-dependent mechanisms are identified by ^∗^, AMPK-independent mechanisms are identified by ^#^, and the studies which did not research the role of AMPK are identified by ^&^.

Organs	Mechanisms							
	TGF-*β*1 signaling pathways					Other signaling pathways		
	Canonical	Noncanonical						
	TGF-*β*1-Smad	MAPK	Wnt	P13K-AKT	mTOR	ROS	Metabolism	Mitochondrial function
Kidney	34^&^, 56^∗^^#^, 60^∗^	33^&^, 34^&^	—	82^&^	88^∗^	94^∗^	32^∗^	—
Heart	25^#^	75^#^	75^∗^	75^∗^, 86^&^	29^∗^	29^∗^, 91^&^, 92^∗^	—	—
Lung	54^∗^, 55^∗^, 57^∗^	54^∗^, 55^∗^	—	—	18^∗^	55^∗^, 57^∗^, 93^∗^	—	18^∗^
Liver	53^&^, 61^∗^	69^∗^, 72^∗^	81^#^	69^∗^, 83^∗^, 85^#^	69^∗^	83^∗^	97^∗^, 100^&^, 101^∗^	—
Adipose	37^∗^	73^&^	—	—	—	—	—	103^#^
Peritoneal	41^∗^^#^	41^∗^^#^	41^∗^^#^	—	—	41^∗^^#^	—	—
Peritendinous	42^∗^	42^∗^	—	—	—	—	—	—
Endometrium	—	70^&^	—	70^&^	—	—	—	—
Skin	—	—	—	84^∗^	—	—	—	—
